# Luteolin alleviates cerebral ischemia/reperfusion injury by regulating cell pyroptosis

**DOI:** 10.1515/med-2024-1063

**Published:** 2024-11-04

**Authors:** Fei Yu, Guangxue Wang, Xingyi Chen, Yanfei Zhang, Cheng Yang, Hui Hu, Liang Wei

**Affiliations:** Department of Neurology, East Hospital, Tongji University School of Medicine, Shanghai, 200120, China; Research Center for Translational Medicine, East Hospital, Tongji University School of Medicine, Shanghai, 200120, China; Department of Medical Department, East Hospital, Tongji University School of Medicine, Shanghai, 200120, China; Department of Neurosurgery, East Hospital, Tongji University School of Medicine, Shanghai, 200120, China; Department of Neurology, East Hospital, Tongji University School of Medicine, 150 Jimo Road, Shanghai, 200120, China; Department of Neurosurgery, East Hospital, Tongji University School of Medicine, 150 Jimo Road, Shanghai, 200120, China

**Keywords:** cerebral ischemia–reperfusion injury, luteolin, MCAO, pyroptosis, high-throughput sequencing

## Abstract

**Objective:**

This study aimed to clarify the roles and underlying mechanisms of luteolin in the progression of cerebral ischemia/reperfusion injury (CIRI).

**Methods:**

A mouse model of CIRI was established using the middle cerebral artery occlusion (MCAO) method, after which luteolin was administered. Subsequently, neuronal apoptosis and pyroptosis were measured and the brain tissues of each group were subjected to RNA sequencing.

**Results:**

Luteolin alleviated MCAO-induced brain infarction, apoptosis, and pyroptosis. RNA sequencing identified 3,379, 2,777, and 3,933 differentially expressed genes (DEGs) in the MCAO vs sham, MCAO vs MCAO + luteolin, and MCAO + luteolin vs sham groups, respectively. The identified DEGs showed enrichment in multiple processes, including pattern specification, forebrain development, anion transport, leukocyte migration, regulation of cell–cell adhesion, and positive regulation of the response to external stimuli, as well as the calcium, PI3K-AKT, JAK-STAT, NF-kappa B, IL-17, cAMP, cGMP-PKG, and Wnt signaling pathways. In addition, *Ccl2* and *Angpt2* interacted more with the other top 30 DEGs with high interaction weights. Finally, RT-qPCR results showed that MCAO induction significantly up-regulated the expression of *Stoml3*, *Eomes*, and *Ms4a15* and down-regulated *Nms*, *Ttr*, and *Avpr1a*; however, luteolin could partially reverse the expression caused by MCAO.

**Conclusion:**

Luteolin can alleviate brain infarction, apoptosis, and pyroptosis in CIRI, and may improve MCAO-induced CIRI by targeting the identified DEGs and their enriched pathways.

## 1 Introduction

Stroke is the second leading cause of death and third leading cause of disability worldwide [1]. It is estimated that over 80% of stroke cases are ischemic stroke (IS), with approximately 14 million cases occurring annually [2,3]. IS is predominantly caused by cerebral artery occlusion, resulting in glucose and oxygen deficiencies in brain cells, triggering oxidative stress, inflammation, apoptosis, and cell death [4,5]. Restoration of the cerebral blood flow perfusion to the ischemic area is the primary treatment strategy for IS [4,6]. However, rapid reperfusion commonly leads to secondary brain tissue damage, known as cerebral ischemia–reperfusion injury (CIRI) [4,6]. Several physiological mechanisms, including atherosclerosis and acute myocardial infarction, promote ischemia and lead to hypoxia and hypoperfusion [7,8]. The obstruction of arterial blood flow leads to hypoxia and dysfunction of the mitochondrial electron transport chain [9]. Reduced ATP production [10] in the mitochondria can induce anaerobic metabolism, sodium potassium pump dysfunction, and ribosome detachment. During reperfusion, the blood flow to the ischemic tissue is restored by providing oxygen through red blood cells; however, due to the low concentration of antioxidants in ischemic cells, the production of reactive oxygen species increases, causing oxidative stress, and promoting endothelial dysfunction, DNA damage, and local inflammatory responses [8]. Currently, intravenous thrombolytic drugs, such as recombinant tissue plasminogen activator, are the only drug treatments approved by the US Food and Drug Administration/European Medicines Agency for patients with acute IS; however, their efficacy is limited by therapeutic time windows, bleeding complications, and single-target approaches [11,12]. As such, it is crucial to develop novel therapeutic strategies for CIRI.

Luteolin (3,4,5,7-tetrahydroxy-flavone) is a flavonoid compound widely found in fruits, herbs, and green vegetables [13,14]. In traditional Chinese medicine, plants rich in luteolin have been extensively used to treat high blood pressure, inflammatory diseases, and cancer, among others [15]. Increasing evidence has shown that luteolin exerts a variety of pharmacological effects, including antioxidant, anti-inflammatory, anticancer, and apoptotic regulatory effects [16,17]. Further, Xu et al. [18] reported that pretreatment with luteolin both *in vitro* and *in vivo* inhibits cell apoptosis via the JPX/miR-146b axis, eventually improving myocardial ischemia–reperfusion injury. A previous study by Jiang et al. [19] also demonstrated that luteolin could suppress inflammation, autophagy, and cell apoptosis via modulation of the ERK/PPARα pathway, thereby alleviating the hepatic cell injury caused by ischemia reperfusion. Another study showed that luteolin protects against renal ischemia–reperfusion injury by regulating pro-inflammatory cytokines, oxidative stress, and cell apoptosis, thereby benefiting kidney transplantation [20]. These reports suggest that luteolin exerts protective effects against ischemia/reperfusion injury. In addition, recent studies have shown that luteolin can provide neuroprotective roles in CIRI [21,22]. For example, Fan et al. [23] illustrated that luteolin-7-*O*-β-d-glucuronide could mitigate CIRI by improving the permeability of the blood–brain barrier. Another investigation showed that luteolin could exhibit neuroprotective effects on CIRI-induced hippocampal inflammation and autophagy by activating PPARγ in rats [24]. However, the molecular mechanisms underlying the protective effects of luteolin against CIRI remain unclear.

Pyroptosis is a form of programmed cell death that plays a key role in host defense against pathogen infections under normal physiological conditions [25]. During this process, the activated NLRP3 inflammasome and procaspase 1 bind to the inflammatory complex via apoptosis-associated spot-like proteins and promote the activation of caspase 1 [25,26]. Activated caspase 1 drives the cleavage of Gasdermin D (GSDMD) to GSDMD-N, thereby processing IL-1β and IL-18, and inducing pyroptosis and corresponding inflammation [25,26]. Xiao et al. [27] further demonstrated that Astragaloside IV alleviates CIRI by activating Nrf2 to inhibit NLRP3 inflammasome-mediated pyroptosis. Another study showed that down-regulation of XBP-1 could protect neurons by inhibiting pyroptosis via action on the classical NLRP3/Caspase-1/GSDMD pathway, thereby mitigating CIRI. Together, these studies revealed that cell pyroptosis plays an essential role in CIRI progression, while modulation of cell pyroptosis could be considered an effective therapeutic strategy for CIRI [28,29]. Furthermore, luteolin has been shown to induce pyroptosis in HT-29 cells by activating the Caspase-1/GSDMD pathway, thereby inhibiting the proliferation of colon cancer cells [30]. However, whether luteolin regulates pyroptosis in CIRI remains unclear.

Given this context, in the present study, we constructed a mouse CIRI model using middle cerebral artery occlusion (MCAO) and used luteolin to treat MCAO-induced mice to explore the role of cell pyroptosis in luteolin-mediated alleviation of CIRI. The brain tissues of mice in different groups were subjected to high-throughput sequencing to uncover other specific molecular mechanisms of luteolin-relieving CIRI. This study provides novel insights into the treatment of CIRI with luteolin, and lays the theoretical foundation for the development of novel therapeutic targets for CIRI.

## 2 Materials and methods

### 2.1 Animals and grouping

A total of 18 male C57BL/6 mice weighing 20 ± 2 g (6–8 weeks) were purchased from the SLAC Laboratory Animal Center of Shanghai (Shanghai, China). All mice were housed under controlled temperature (24 ± 2°C) and humidity (50  ± 5%), with a 12 h light/dark cycle, with access to food and water provided *ad libitum*. After acclimatization for 7 days, 18 mice were randomly and equally divided into three groups (*n* = 6 per group): sham, MCAO, and MCAO + luteolin.

Mice in the MCAO and MCAO + luteolin groups underwent induction of CIRI *in vivo* using MCAO. In brief, mice were anesthetized with 3% pentobarbital sodium, and an incision was made in the midline of the neck to expose the left common carotid artery, external carotid artery, and internal carotid artery. Subsequently, a 3.0 nylon suture was advanced from the external carotid artery to the internal carotid artery until the middle cerebral artery was blocked. After 1 h, the suture was retracted to initiate reperfusion, and the wound was closed. The mice in the sham group underwent sham operation without ligation. Mice in the MCAO + luteolin group were intraperitoneally injected with 5 mg/kg luteolin (Shanghai Yuanye Bio-Technology Co., Ltd, Shanghai, China) following suture removal. After 24 h, all mice were euthanized by CO_2_ inhalation, and their brain tissue was collected.


**Ethical approval:** The research related to animal use has been complied with the National Medical Advisory Committee guidelines using procedures approved by the Institutional Animal Care and Use Committee at East Hospital, Tongji University School of Medicine.

### 2.2 2,3,5-Triphenyltetrazolium chloride (TTC) staining

Brain infarction in different groups was assessed using TTC staining. In brief, after the mice were euthanized, the cerebrum was extracted and maintained at −20°C. Subsequently, 2 mm coronal sections were obtained and immersed in 0.5% TTC reagent (Shanghai Yuanye Biotechnology Co. Ltd, Shanghai, China) for 30 min before fixation in 4% paraformaldehyde. The brain infarct volumes of the mice were measured using ImageJ software.

### 2.3 TUNEL staining

Neuronal apoptosis in different groups was detected using TUNEL staining, as previously described [31]. In brief, the collected brain tissues were fixed, embedded, and prepared into 4 µm slices. The slices were then deparaffinized and rehydrated, and endogenous peroxidase was blocked. Subsequently, the slices were used to evaluate neuronal apoptosis using the DAB (SA-HRP) TUNEL Cell Apoptosis Detection Kit (Servicebio, Wuhan, China), following the manufacturer’s protocols. Apoptosis in different groups was measured under a microscope.

### 2.4 Real-time quantitative polymerase chain reaction (qPCR)

Total RNA was extracted from the brain tissues of different groups using RNAiso Plus (Takara, Tokyo, Japan), and reverse transcribed into cDNA using PrimeScript RT Master Mix (Takara). The RT-qPCR assay was conducted using 2× Universal SYBR Green Fast qPCR Mix (ABclonal, Wuhan, China). The sequences of all primers are shown in Table 1. GAPDH was used as an internal reference. The relative mRNA expressions of related genes were calculated using the 2^−ΔΔCt^ method.

**Table 1:** Sequences of all primers

Primer	Sequence (5′–3′)
TNF-α-F	CTGAACTTCGGGGTGATCGG
TNF-α-R	GGCTTGTCACTCGAATTTTGAGA
IL-1β-F	TGCCACCTTTTGACAGTGATG
IL-1β-R	TGATGTGCTGCTGCGAGATT
NLRP3-F	ATTACCCGCCCGAGAAAGG
NLRP3-R	TCGCAGCAAAGATCCACACAG
Caspase1-F	ACAAGGCACGGGACCTATG
Caspase1-R	TCCCAGTCAGTCCTGGAAATG
GSDMD-F	CCATCGGCCTTTGAGAAAGTG
GSDMD-R	ACACATGAATAACGGGGTTTCC
GAPDH-F	GGTGAAGGTCGGTGTGAACG
GAPDH-R	CTCGCTCCTGGAAGATGGTG
Stoml3-F	GATTCACCGGAGAAACTGGAG
Stoml3-R	TCCATACTGAGATTGGGAAGGT
Eomes-F	GCGCATGTTTCCTTTCTTGAG
Eomes-R	GGTCGGCCAGAACCACTTC
Ms4a15-F	GCGCCCAGAGCTACTCAAC
Ms4a15-R	GTGGATGAGACCGATCAGGAT
Scgn-F	ATGGACAACGCACGCAGAAA
Scgn-R	TCCTCAGTGCCAGATTTTGCC
Nms-F	CCAATCCTGTTCATCTACTGCTT
Nms-R	CGGGAGAATCAGCTAAAGGTG
Ttr-F	CTGCTGTAGACGTGGCTGTAA
Ttr-R	CTTCCAGTACGATTTGGTGTCC
Avpr1a-F	GCTGGCGGTGATTTTCGTG
Avpr1a-R	GCAAACACCTGCAAGTGCT
Nkx2-1-F	ATGAAGCGCCAGGCTAAGG
Nkx2-1-R	GGTTTGCCGTCTTTGACTAGG

### 2.5 Western blot

Total protein was isolated from the brain tissues of different groups using RIPA lysis buffer (Beyotime Biotechnology, Shanghai, China) and quantified using a BCA protein assay kit (Beyotime Biotechnology). Subsequently, protein samples (20 μg) were separated by 10% SDS-PAGE and transferred to PVDF membranes. After being blocked with 5% skim milk at 37°C for 2 h, membranes were incubated with the following primary antibodies: anti-MMP9 (1:1,000, 10375-2-AP; Proteintech), anti-NLRP3 (1:2,000, 68102-1-Ig; Proteintech), anti-IL-6 (1:1,000, 26404-1-AP; Proteintech), anti-IL-1β (1:800, A16288; Abclonal), and anti-GAPDH (1:5,000, 60004-1-Ig; Proteintech) at 4°C overnight, before incubation with the relevant secondary antibody (1:10,000; Proteintech) at 37°C for 1 h. After washing, the blots were visualized using an ECL chemiluminescence kit (Beyotime), and quantified using ImageJ software.

### 2.6 Library construction and sequencing

RNA from the sham, MCAO, and MCAO + luteolin groups was sent to Yanzai Biotechnology (Shanghai) Co. Ltd (Shanghai, China) for RNA sequencing. In brief, RNA samples were checked by RNase-free agarose gel electrophoresis and quantified using a NanoDrop 2000. Subsequently, sequencing transcriptome libraries were prepared using the TruSeq™ RNA Sample Prep Kit (Illumina, San Diego, CA, USA), and RNA sequencing was carried out using the NovaSeq 6000 S4 Reagent Kit v1.5 (300 cycles) (Illumina, San Diego, CA, USA) and the Illumina NovaSeq2500 (Illumina, San Diego, CA, USA) system, following the manufacturer’s instructions.

### 2.7 Data analysis and bioinformatic analysis

Raw sequencing reads were subjected to optimization processes, such as de-sequencing splices, quality splicing, short sequence filtering, and removal of ribosomal contamination. The processed reads were compared to the mouse reference genome (GRCm39.106). Subsequently, differentially expressed genes (DEGs) among the different groups were identified based on the following thresholds: adjusted *P* value <0.05 and |log_2_ fold change (FC)|  ≥ 1. The identified DEGs were then subjected to gene ontology (GO) and Kyoto Encyclopedia of Genes and Genomes (KEGG) pathway enrichment analyses, and protein–protein interaction (PPI) networks were constructed using the STRING tool.

### 2.8 Statistical analysis

Data are shown as mean ± standard deviation, and analyzed by GraphPad Prism 8.4.2. Differences between the groups were evaluated using one-way analysis of variance followed by Tukey’s *post hoc* test. Statistical significance was set at *P* < 0.05.

## 3 Results

### 3.1 Luteolin alleviates MCAO-induced brain infarction, cell apoptosis, and pyroptosis

**Figure 1:**
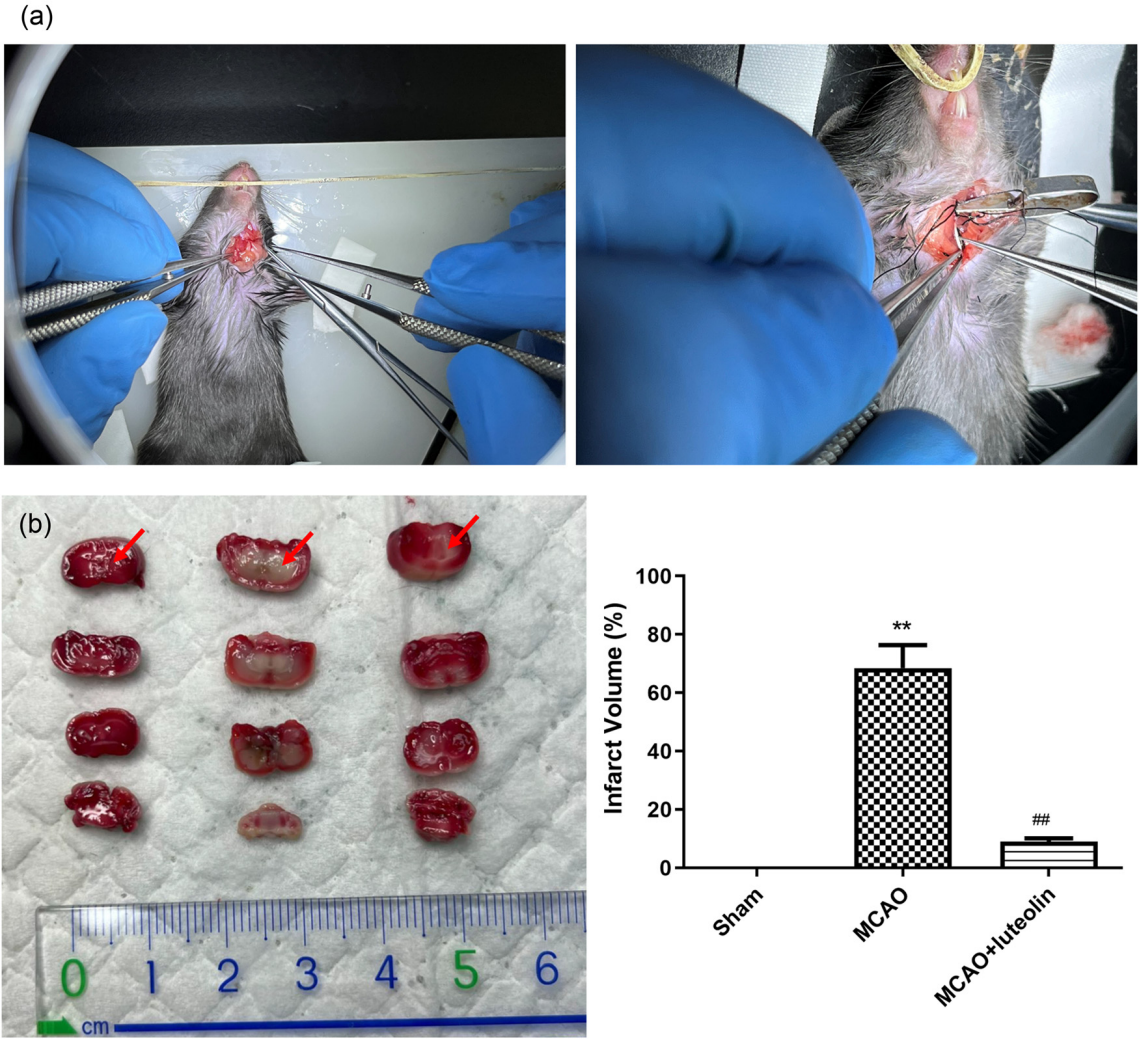
Luteolin alleviates MCAO-induced brain infarction. (a) A surgical diagram outlining MCAO model establishment. (b) Cerebral infarction in the MCAO model after luteolin treatment evaluated by TTC staining. **P < 0.01 vs sham group; ^##^ *P* < 0.01 vs MCAO group.

To clarify the role of luteolin in CIRI progression, a mouse model of CIRI was established using MCAO, after which luteolin was applied, as shown in Figure 1a. The brain infarct volumes in the different groups were then assessed. Compared to the sham group, MCAO induction significantly increased the brain infarct volume (*P* < 0.01), whereas luteolin administration reduced the brain infarct volume induced by MCAO (*P* < 0.01, Figure 1b and c), indicating that luteolin could alleviate brain infarction induced by MCAO, thereby exerting a protective role against CIRI.

**Figure 2:**
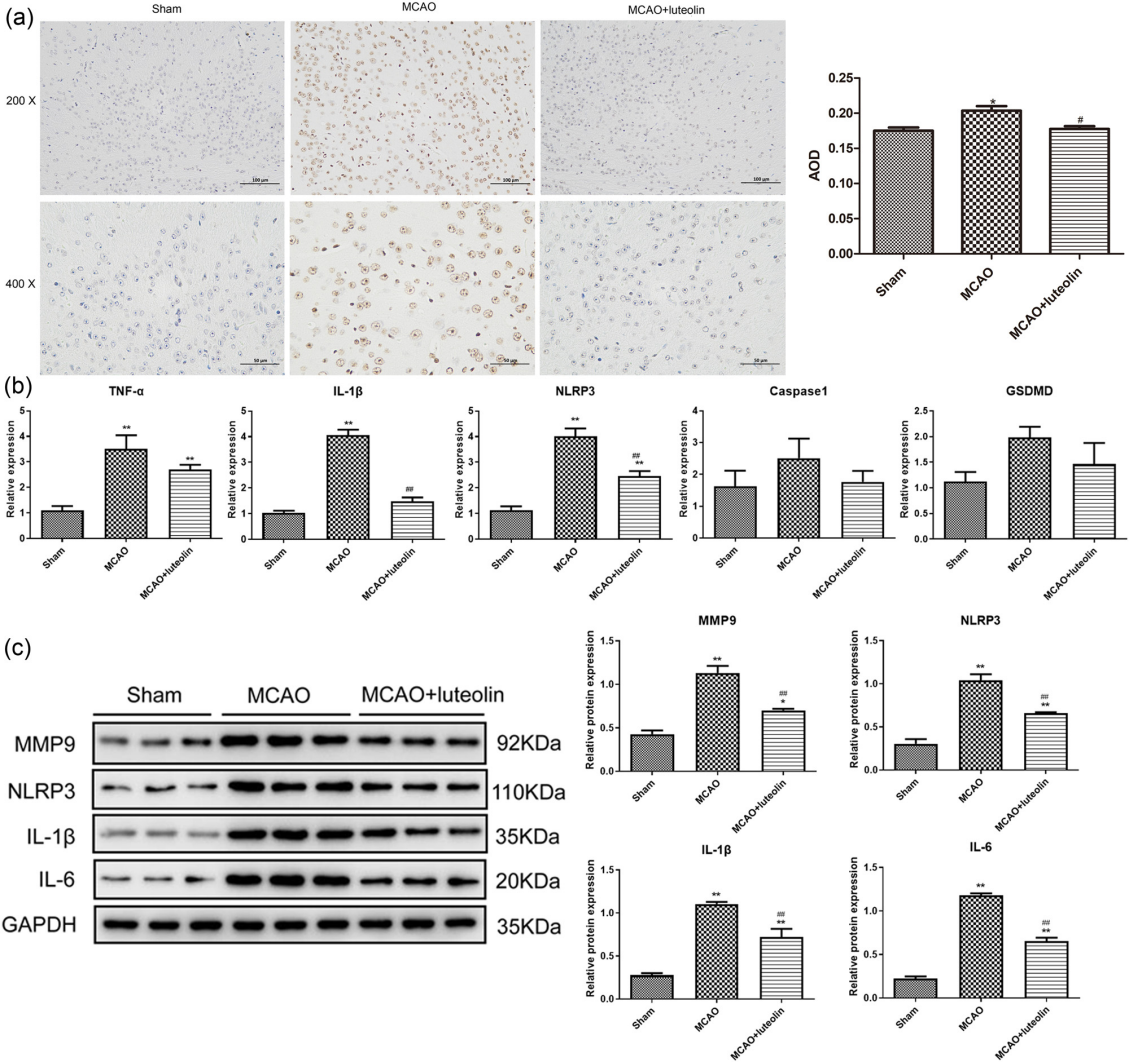
Luteolin alleviates MCAO-stimulated cell apoptosis and pyroptosis. (a) Cell apoptosis in the brain tissues of MCAO mice after luteolin treatment assessed using TUNEL staining. (b) The mRNA expression levels of *TNF-α*, *IL-1β*, *NLRP3*, *Caspase-1*, and *GSDMD* in brain tissues of MCAO mice after luteolin treatment measured by RT-qPCR. (c) The protein expression of MMP9, NLRP3, IL-1β, and IL-6 in brain tissues of MCAO mice after luteolin treatment detected using western blot. *P < 0.05, **P < 0.01 vs sham group; ^#^ *P* < 0.05, ^##^ *P* < 0.01 vs MCAO group.

Herein, we examined the effects of luteolin on apoptosis and pyroptosis. Apoptosis was significantly higher in the MCAO group than in the sham group (*P* < 0.05), whereas luteolin treatment inhibited MCAO-induced apoptosis (*P* < 0.05, Figure 2a). Subsequently, the expression levels of inflammatory cytokines (TNF-α, IL-1β, and IL-6) and pyroptosis-related factors (NLRP3, Caspase-1, GSDMD, and MMP9) were analyzed by RT-qPCR and western blot. RT-qPCR showed significant up-regulation of the mRNA expressions of *TNF-α*, *IL-1β*, and *NLRP3* in MCAO mice compared with sham mice (*P* < 0.05); while luteolin administration reversed this expression induced by MCAO (*P* < 0.05, Figure 2b). However, no significant differences were found in the mRNA expression levels of Caspase-1 and GSDMD among the sham, MCAO, and MCAO + luteolin groups (*P* > 0.05, Figure 2b). Furthermore, western blot analysis showed significant increases in the protein levels of MMP9, NLRP3, IL-1β, and IL-6 following MCAO induction relative to the sham mice (*P* < 0.05), which was partially abrogated by luteolin (*P* < 0.05, Figure 2c). These results indicate that luteolin can mitigate MCAO-induced apoptosis and partially restore the expression of pro-inflammatory cytokines and pyroptosis-related factors induced by MCAO.

### 3.2 Identification of DEGs among different groups

**Figure 3:**
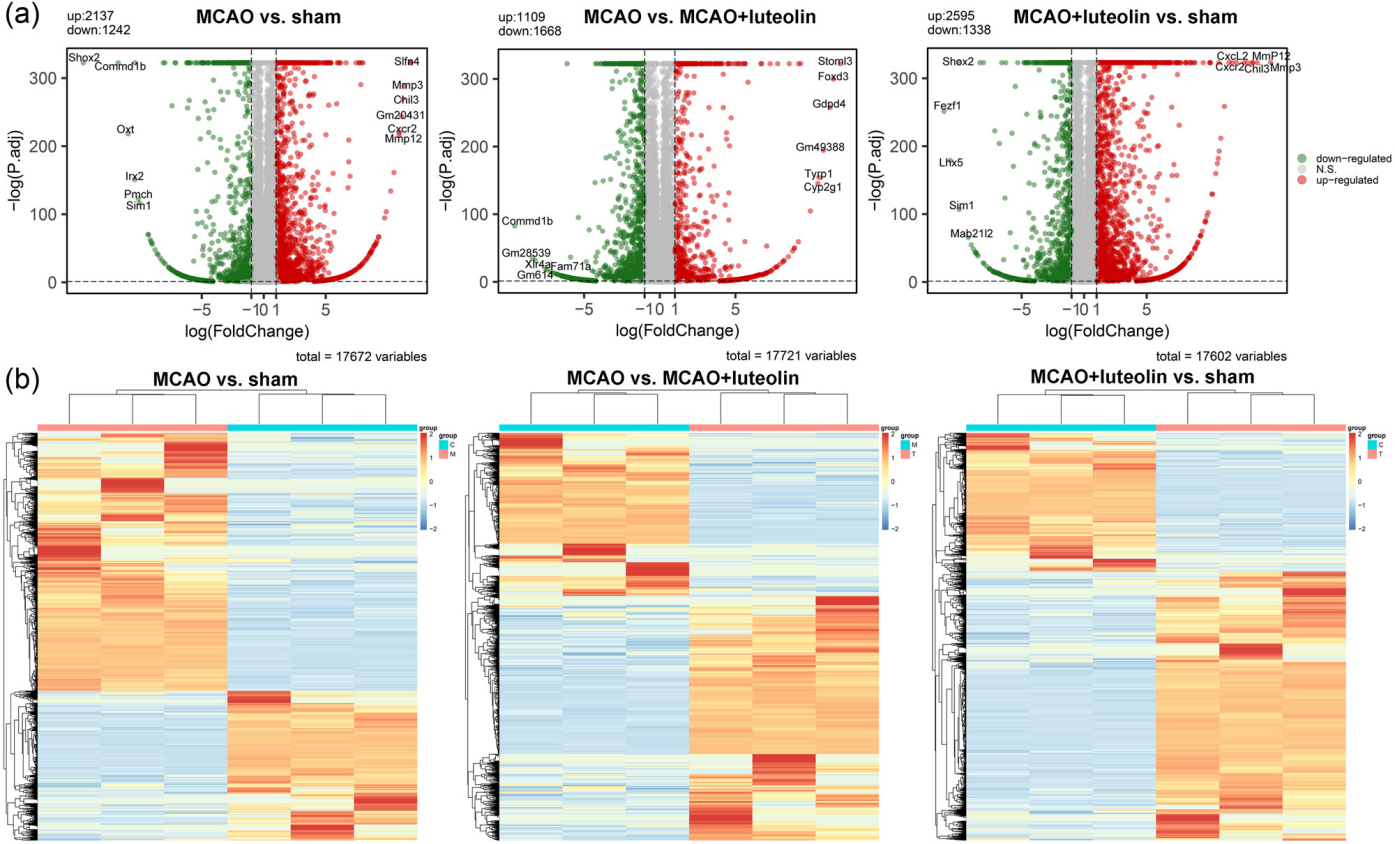
Identification of DEGs among different groups. (a) Volcano plots of DEGs in the comparison groups of MCAO vs sham, MCAO vs MCAO + luteolin, and MCAO + luteolin vs sham. (b) Bidirectional hierarchical clustering heatmaps of the identified DEGs in the different comparison groups of MCAO vs sham, MCAO vs MCAO + luteolin, and MCAO + luteolin vs sham.

To elucidate the potential molecular mechanisms through which luteolin protects against CIRI, the total RNA of brain tissues in the sham, MCAO, and MCAO + luteolin groups was subjected to RNA sequencing. Based on the established criteria (adjusted *P* value < 0.05 and |log_2_ FC|  ≥ 1), 3,379 (2,137 up-regulated and 1,242 down-regulated in MCAO), 2,777 (1,109 up-regulated and 1,668 down-regulated in MCAO), and 3,933 (2,595 up-regulated and 1,338 down-regulated in MCAO + luteolin) DEGs were identified in the comparison of MCAO vs sham, MCAO vs MCAO + luteolin, and MCAO + luteolin vs sham, respectively (Figure 3a). In the comparison between MCAO and sham, the top five up-regulated DEGs were *Slfn4*, *Mmp3*, *Chil3*, *Gm20431*, and *Cxcr2*, while the top five down-regulated DEGs were *Shox2*, *Commd1b*, *Oxt*, *Irx2*, and *Pmch* (Table 2). In the comparison between the MCAO and MCAO + luteolin, the top five up-regulated DEGs were *Stoml3*, *Foxd3*, *Gdpd4*, *Gm49388*, and *Tyrp1*, and the top five down-regulated DEGs were *Commd1b*, *Gm28539*, *Xlr4a*, *Fam71a*, and *Gm614* (Table 1). In addition, *Mmp3, Mmp12, Chil3, Cxcl2*, and *Cxcr2* were the top five up-regulated DEGs, whereas *Fezf1, Lhx5, Shox2, Sim1*, and *Mab21l2* were the top five down-regulated DEGs in the MCAO + luteolin group compared to the sham group (Table 1). In addition, the bidirectional hierarchical clustering heatmaps of the identified DEGs in the different comparison groups are displayed in Figure 3b, which show that the identified DEGs in the different comparison groups could significantly distinguish the two different groups.

**Table 2:** Top five DEGs with up-regulation and down-regulation in the comparison groups of MCAO vs sham, MCAO + luteolin vs MCAO, and MCAO + luteolin vs sham

DEGs	Comparison					
	MCAO vs sham		MCAO + luteolin vs MCAO		MCAO + luteolin vs sham	
	Up-regulation (log_2_ FC)	Down-regulation (log_2_ FC)	Up-regulation (log_2_ FC)	Down-regulation (log_2_ FC)	Up-regulation (log_2_ FC)	Down-regulation (log_2_ FC)
1	Slfn4 (11.9)	Shox2 (−14.6)	Stoml3 (11.9)	Commd1b (−9.6)	Mmp3 (14.9)	Fezf1 (−11.2)
2	Mmp3 (11.8)	Commd1b (−11.7)	Foxd3 (11.5)	Gm28539 (−8.4)	Mmp12 (13.8)	Lhx5 (−10.7)
3	Chil3 (11.4)	Oxt (−10.9)	Gdpd4 (11.3)	Xlr4a (−8.2)	Chil3 (13.5)	Shox2 (−10.0)
4	Gm20431 (11.2)	Irx2 (−10.4)	Gm49388 (10.8)	Fam71a (−7.5)	Cxcl2 (13.5)	Sim1 (−9.9)
5	Cxcr2 (11.1)	Pmch (−10.4)	Tyrp1 (10.5)	Gm614 (−7.4)	Cxcr2 (13.1)	Mab21l2 (−9.3)

### 3.3 GO term analysis of the identified DEGs in the different comparison groups

DEGs identified in the different comparison groups were subjected to GO term analysis, including biological processes (BP), cellular components (CC), and molecular functions (MF). As shown in Tables S1 and S2, the up-regulated DEGs in the comparison of MCAO vs sham were enriched in “positive regulation of response to external stimulus,” “regulation of immune effector process,” “cytokine-mediated signaling pathway,” “negative regulation of immune system process,” and “leukocyte migration”; while the down-regulated DEGs were enriched in “adenylate cyclase-modulating G protein-coupled receptor signaling pathway,” “regulation of membrane potential,” “hormone transport,” “cilium movement,” and “locomotory behavior” in BP. In CC, the up-regulated DEGs between the sham and MCAO groups largely participated in “collagen-containing extracellular matrix,” “apical part of cell,” “receptor complex,” “membrane microdomain,” and “membrane raft”; while the down-regulated DEGs were associated with “synaptic membrane,” “motile cilium,” “transmembrane transporter complex,” “transporter complex,” and “ion channel complex” (Tables S1 and S2). At the MF level, the up-regulated DEGs between the sham and MCAO groups were closely related to “endopeptidase activity,” “cytokine activity,” “cytokine receptor binding,” “cell adhesion molecule binding,” and “sulfur compound binding”; while the decreased DEGs were correlated with “channel activity,” “passive transmembrane transporter activity,” “ion channel activity,” “gated channel activity,” and “metal ion transmembrane transporter activity” (Tables S1 and S2).

In the comparison between the MCAO and MCAO + luteolin groups, the up-regulated DEGs were significantly enriched in the pattern specification process “pattern specification process,” “forebrain development,” “anion transport,” “visual system development,” and “sensory system development” in BP; “apical part of cell,” “synaptic membrane,” “transporter complex,” “transmembrane transporter complex,” and “collagen-containing extracellular matrix” in CC; and “channel activity,” “passive transmembrane transporter activity,” “ion channel activity,” “metal ion transmembrane transporter activity,” and “anion transmembrane transporter activity” in MF (Table S3). Further, the down-regulated DEGs were correlated with “leukocyte migration,” “regulation of cell–cell adhesion,” “positive regulation of response to external stimulus,” and “leukocyte cell–cell adhesion in BP; “collagen-containing extracellular matrix,” “receptor complex,” “secretory granule,” “apical part of cell,” and “membrane raft” in CC; and “cytokine receptor binding,” “G protein-coupled receptor binding,” “channel activity,” “cytokine activity,” and “enzyme inhibitor activity” in MF (Table S4).

In the comparison of MCAO + luteolin vs sham, up-regulated DEGs were significantly enriched in “positive regulation of response to external stimulus,” “leukocyte migration,” “positive regulation of defense response,” “cytokine-mediated signaling pathway,” and “regulation of immune effector process” in BP; “collagen-containing extracellular matrix,” “membrane microdomain,” “membrane raft,” and “receptor complex” in CC; and “cytokine activity,” “immune receptor activity,” and “cytokine binding” in MF (Table S5). Furthermore, the identified down-regulated DEGs were closely related to “neuropeptide signaling pathway,” “catecholamine transport,” “forebrain development,” and “positive regulation of cytosolic calcium ion concentration” in BP, “ion channel complex,” “transporter complex,” and “transmembrane transporter complex” in CC, and “gated channel activity,” “ion channel activity,” and “G protein-coupled peptide receptor activity” in MF (Figure S6).

### 3.4 KEGG pathway analysis of the identified DEGs in the different comparison groups

**Figure 4:**
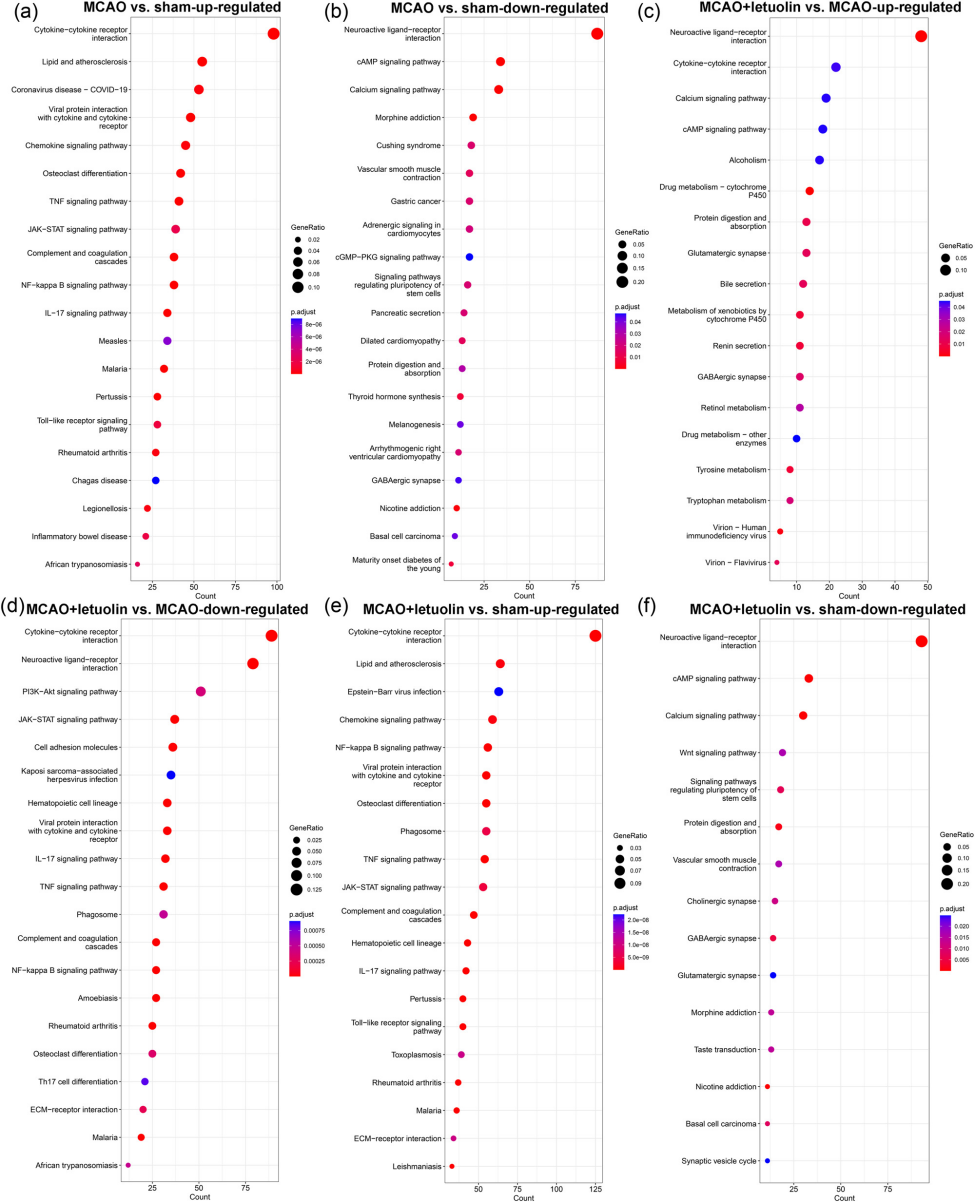
KEGG pathways of the identified DEGs in the different comparison groups. (a) KEGG analysis of the up-regulated DEGs between the sham and MCAO groups. (b) KEGG analysis of the down-regulated DEGs between the sham and MCAO groups. (c) KEGG analysis of the up-regulated DEGs between the MCAO and MCAO + luteolin groups. (d) KEGG analysis of the down-regulated DEGs between the MCAO and MCAO + luteolin groups. (e) KEGG analysis of the up-regulated DEGs between the sham and MCAO + luteolin groups. (f) KEGG analysis of the down-regulated DEGs between the sham and MCAO + luteolin groups.

KEGG analysis was further conducted to study the enrichment pathways of the identified DEGs. This analysis revealed that the up-regulated DEGs in the MCAO group compared to the sham group were primarily involved in “chemokine signaling pathway,” “TNF signaling pathway,” “JAK-STAT signaling pathway,” “NF-kappa B signaling pathway,” “IL-17 signaling pathway,” and “Toll-like receptor signaling pathway” (Figure 4a); while the down-regulated DEGs in the MCAO group compared to the sham group were related to “cAMP signaling pathway,” “calcium signaling pathway,” “cGMP-PKG signaling pathway,” and “neuroactive ligand–receptor interaction” (Figure 4b). The identified DEGs between the MCAO and MCAO + luteolin groups were significantly enriched in “neuroactive ligand–receptor interaction,” “cAMP signaling pathway,” “calcium signaling pathway,” “PI3K-Akt signaling pathway,” “JAK-STAT signaling pathway,” “TNF signaling pathway,” “IL-17 signaling pathway,” “NF-kappa B signaling pathway,” and “Th17 cell differentiation” (Figure 4c and d). Additionally, for the identified DEGs between the MCAO + luteolin and sham groups, the pathways enriched in up-regulated DEGs included the “chemokine signaling pathway,” “Toll-like receptor signaling pathway,” “TNF signaling pathway,” “IL-17 signaling pathway,” and “NF-kappa B signaling pathway” (Figure 4e); while the pathways enriched in the down-regulated DEGs included “neuroactive ligand–receptor interaction,” “cAMP signaling pathway,” “calcium signaling pathway,” and “Wnt signaling pathway” (Figure 4f).

### 3.5 Analysis of the constructed PPI networks

**Figure 5:**
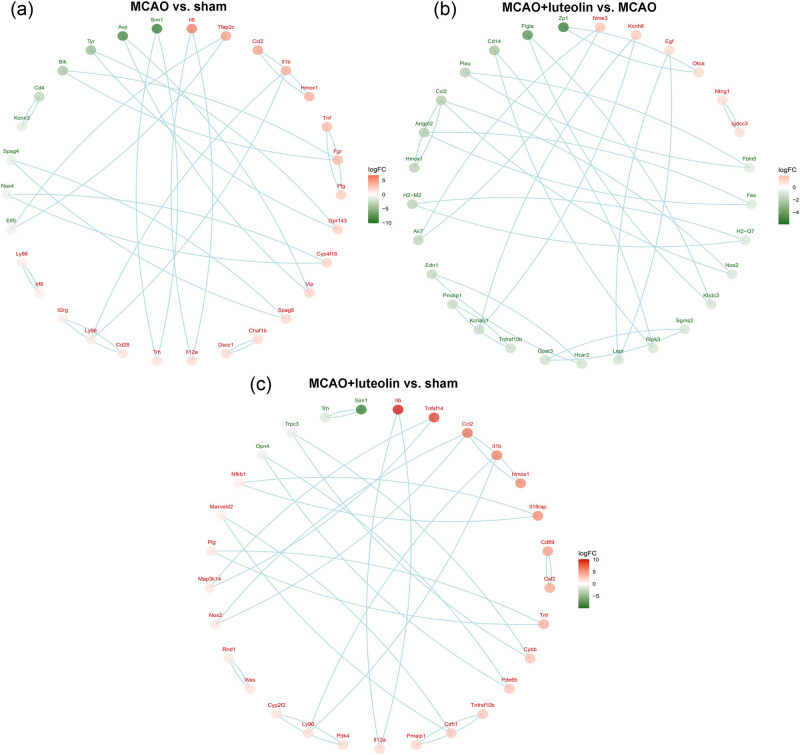
Analysis of the constructed PPI networks. (a) The PPI network of the top 30 DEGs between the sham and MCAO groups. (b). The PPI network of the top 30 DEGs between the MCAO and MCAO + luteolin groups. (c) The PPI network of the top 30 DEGs between the sham and MCAO + luteolin groups.

DEGs identified in the different comparison groups were used to construct PPI networks using the STRING database. The top 30 DEGs were sorted based on their interaction weights from the highest to lowest among the three comparison groups (MCAO vs sham, MCAO vs MCAO + luteolin, and MCAO + luteolin vs sham). The top 30 DEGs with higher interaction weights between the sham and MCAO groups are presented in Figure 5a; in this comparison the top three up-regulated DEGs were *IL6*, *Tfap2c*, and *Ccl2*, while the top three down-regulated DEGs were *Sim1*, *Avp*, and *Tyr*. In the PPI network of the identified DEGs in the comparison of MCAO vs MCAO + letuolin, the top three up-regulated DEGs were *Nme3*, *Kcnh6*, and *Egf*, while the top three down-regulated DEGs were *Zp1*, *Figia*, and *Cd14*; among which *Ccl2* and *Angpt2* presented more interactions with other DEGs (Figure 5b). Additionally, in the PPI network of the identified DEGs in the comparison of MCAO + letuolin vs sham, the top three up-regulated DEGs were *Il6*, *Tnfsf14*, and *Ccl2*, while the top three down-regulated DEGs were *Sim1*, *Trh*, and *Trpc3*, among which *Ccl2* interacted more other DEGs (Figure 5c).

### 3.6 Validation of the sequencing results by RT-qPCR

**Figure 6:**
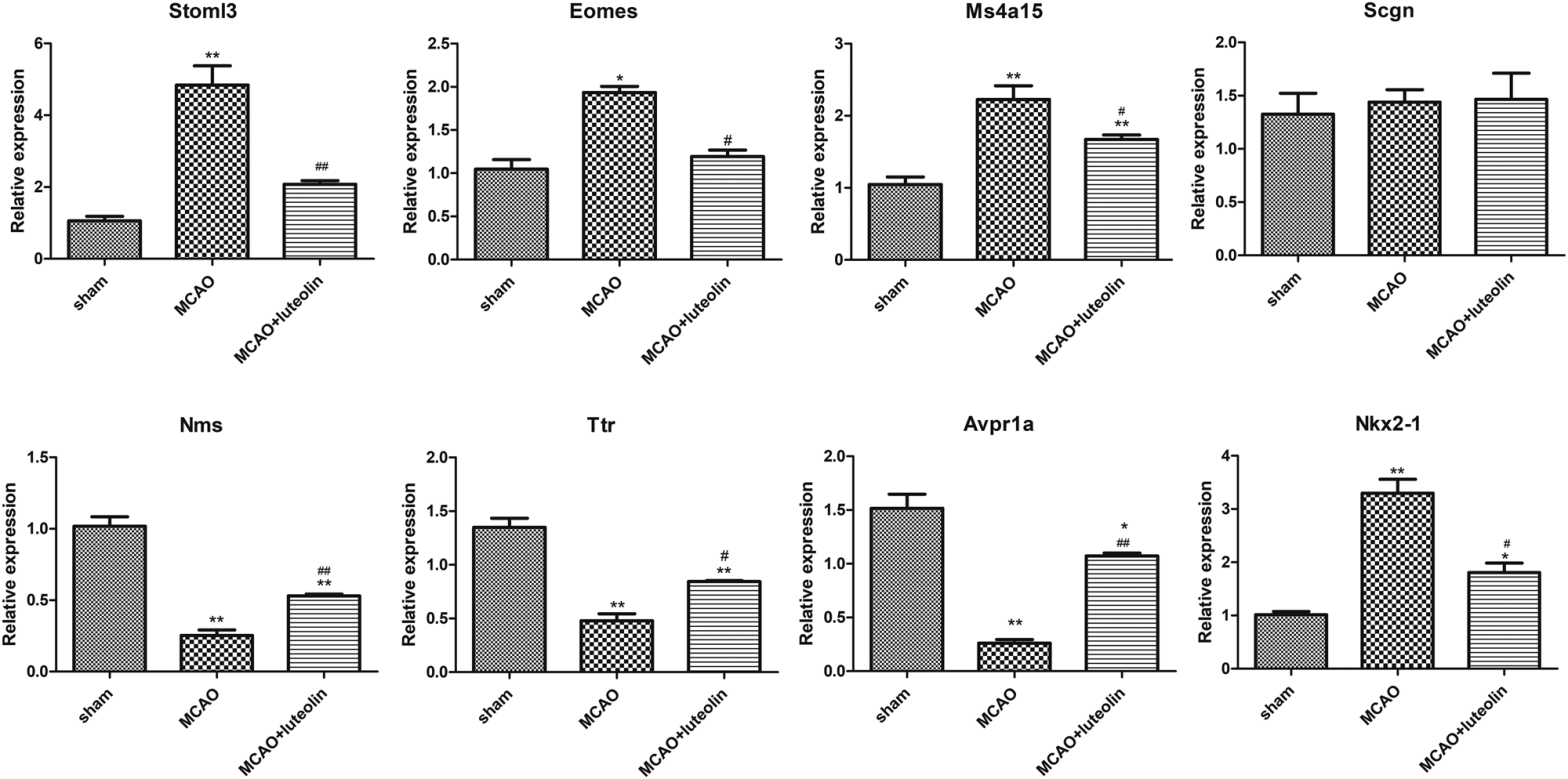
Validation of the RT-qPCR sequencing data. The mRNA expression levels of *Stoml3*, *Eomes*, *Ms4a15*, *Scgn*, *Nms*, *Ttr*, *Avpr1a*, and *Nkx2-1* in the different groups. *P < 0.05, **P < 0.01 vs sham group; ^#^ *P* < 0.05, ^##^ *P* < 0.01 vs MCAO group.

Finally, DEGs with significant fold changes and different expression trends in different groups were selected for RT-qPCR validation. In the current study, *Stoml3*, *Eomes*, *Ms4a15*, and *Scgn* (up-regulated by MCAO but down-regulated after luteolin treatment), as well as *Nms*, *Ttr*, *Avpr1a*, and *Nkx2-1* (down-regulated in MCAO but up-regulated after luteolin treatment) were chosen for validation by RT-qPCR. Compared to the sham group, the mRNA expressions of *Stoml3*, *Eomes*, and *Ms4a15* were significantly up-regulated in MCAO-induced mice (*P* < 0.05), and down-regulated after luteolin treatment (*P* < 0.05, Figure 6). However, *Scgn* mRNA expression showed no significant differences among the sham, MCAO, and MCAO + luteolin groups (*P* > 0.05; Figure 6). Analysis of *Nms*, *Ttr*, and *Avpr1a* revealed marked decreases in expression after MCAO induction compared to the sham group (*P* < 0.05), which was enhanced by luteolin administration (*P* < 0.05, Figure 6). However, *Nkx2-1* expression was significantly up-regulated by MCAO (*P* < 0.05), but was down-regulated by luteolin (*P* < 0.05, Figure 6). After comparison with the sequencing data, we found that the expression tendencies of *Stoml3*, *Eomes*, *Ms4a15*, *Nms*, *Ttr*, and *Avpr1a* determined by RT-qPCR were consistent with those detected by RNA sequencing, yielding a consistency rate of 70% between the RT-qPCR and RNA sequencing results, indicating a relatively high reliability of RNA sequencing.

## 4 Discussion

CIRI is a complicated disease associated with high disability and mortality rates that can seriously impact the health and life of patients [32]. Luteolin, a flavonoid compound, has been shown to freely penetrate the blood–brain barrier, providing neuroprotection against brain injury [33]. However, the role of luteolin in CIRI has not been thoroughly investigated. Therefore, the present study comprehensively clarified the potential mechanisms underlying the role of luteolin in CIRI progression. To investigate the mechanisms of action of luteolin in CIRI, a mouse model of CIRI was established by MCAO and then treated with luteolin. The MCAO model is one of the models that most closely simulate human IS, and is widely used in IS research [34]. Our study showed that MCAO induction caused brain infarction in mice and that luteolin significantly alleviated this phenomenon. A previous study by Tan et al. reported that luteolin decreased the brain infarct area and neuronal injury in an MCAO rat model [21]. Liu et al. further revealed that luteolin could decrease the cerebral edema and infarction volumes, thereby alleviating neurological injury [22]. These results indicate that luteolin could reduce the brain infarct area in MCAO-induced mice, thereby alleviating neuronal injury.

Apoptosis and pyroptosis are the two most common forms of programmed cell death. Cell apoptosis is critical for disease development and tissue homeostasis, and has an important impact on CIRI progression [35,36]. A previous study by Xie et al. [37] showed that cellular apoptosis was exacerbated in rat brain tissues following MCAO, whereas dihydromyricetin alleviated the apoptosis induced by MCAO. Our study further showed that MCAO significantly enhanced apoptosis, which was alleviated by luteolin treatment. Our results are consistent with those of a previous study [22]. Pyroptosis is caspase-1-dependent and inflammation-related programmed cell death pathway that plays a role in CIRI pathogenesis [25]. Our study found that MCAO up-regulated the expression of TNF-α, IL-1β, IL-6, MMP9, and NLRP3; whereas luteolin could reverse these increases. TNF-α, IL-1β, and IL-6 are pro-inflammatory cytokines, closely associated with pyroptosis. Li et al. [38] previously demonstrated that isoliquiritin could improve neuronal survival and morphology, and reduce pyroptosis-related neuronal cell death by decreasing the levels of TNF-α, IL-1β, IL-6, cleaved Caspase-1, NLRP3, and GSDMD-N, thereby ameliorating depression. MMP9 is a component of the extracellular matrix that increases after stroke. This disturbance is associated with disruption of the blood–brain barrier, an increased risk of bleeding complications, and worsening outcomes [39]. A previous study showed that MMP9 is highly expressed in human lung transplantation and rat lung ischemia–reperfusion tissues, and could contribute to lung ischemia–reperfusion injury by activating cell pyroptosis [40]. The NLRP3 inflammasome participates in the inflammatory response, and its assembly and activation lead to the Caspase-1-dependent release of pro-inflammatory cytokines (IL-1β and IL-18) [41]. It has further been reported that the role of the NLRP3 inflammasome in CIRI primarily involves the release and pyroptosis of NLRP3 inflammasome-dependent cytokines, indicating that NLRP3 may be a therapeutic target in CIRI [42]. Based on these results, we speculate that luteolin may suppress cell apoptosis and pyroptosis by down-regulating the expression of TNF-α, IL-1β, IL-6, MMP9, and NLRP3, thereby improving CIRI.

To further understand the molecular mechanisms by which luteolin improves CIRI, brain tissue samples were subjected to high-throughput sequencing. This analysis identified 3,379, 2,777, and 3,933 DEGs in the MCAO vs sham, MCAO vs MCAO + luteolin, and MCAO + luteolin vs sham comparison groups, respectively. The increased DEGs in the MCAO model after luteolin treatment was predominantly enriched in BP of “pattern specification process,” “forebrain development,” and “anion transport”; while the down-regulated DEGs in the MCAO model after luteolin treatment were primarily enriched in BP of “leukocyte migration,” “regulation of cell–cell adhesion,” and “positive regulation of response to external stimulus.” This pattern specification process can modulate cell differentiation during development and early patterning of the mesoderm [43]. Li et al. also found that the pattern specification process was primarily enriched in DEGs in hepatic I/R injury [44]. Anion transport critically affects CIRI development [45,46]. Dai et al. showed that chloride (Cl^–^) efflux-regulated cerebral circulation after neonatal hypoxia–ischemia can contribute to brain injury [45]. Kohut et al. further showed that anion transport inhibitors inhibit ischemic brain injury [46]. Leukocytes contribute to tissue damage following cerebral ischemia and can adhere to the wall of pial postcapillary venules at the start of reperfusion and then migrate to the brain tissue [47]. Cell adhesion plays a critical role in the assembly of individual cells into tissues [48]. Previous studies have shown that cell adhesion is involved in CIRI [49,50]. For example, leukocyte–endothelial cell adhesion occurs following CIRI [50]. In addition, we showed that the DEGs in the MCAO model after luteolin treatment were related to positive regulation of the response to external stimuli. Similarly, Liu et al. found that the DEGs in celastrol-treated MCAO mice were enriched in the positive regulation of responses to external stimuli [51]. These reports, together with the results of our study, suggest that the processes of pattern specification, forebrain development, anion transport, leukocyte migration, regulation of cell–cell adhesion, and positive regulation of responses to external stimuli might exert critical functions in the recovery of CIRI.

In addition, KEGG analysis showed a close association of the identified DEGs with the calcium, PI3K-AKT, JAK-STAT, NF-kappa B, IL-17, cAMP, cGMP-PKG, and Wnt signaling pathways. Calcium homeostasis plays a vital role in maintaining excitation–contraction coupling, and drugs associated with the calcium-signaling pathway have been reported to alleviate cerebrovascular diseases, including CIRI [52]. PI3K-AKT signaling pathway is also known as a critical pathway that facilitates the survival of ischemic nerve cells in CIRI [53]. As such, this pathway is considered an effective target for CIRI therapy [54]. The JAK-STAT pathway mediates neuroinflammation, and is involved in the regulation of physiological activities such as cell proliferation, differentiation, and apoptosis, which play important roles in CIRCI [55]. Li et al. previously reported that quercetin could facilitate the M2 polarization of microglia/macrophages by regulating the PI3K/Akt/NF-κB signaling pathway, thereby ameliorating CIRI [56]. A previous study also showed that papaverine exhibited anti-inflammatory immunomodulatory effects on CIRI via the IL-17 pathway, with the activation of IL-23, NF-κB, RANKL, and p38MAPK [57]. Wnt signaling pathway includes canonical Wnt/β-catenin, non-canonical Wnt/PCP, and Wnt/Ca2^+^ pathways, which are pivotal for CIRI [58]. Chen et al. [59] further demonstrated that the suppression of LMP2 could improve the blood–brain barrier dysfunction induced by IR through activation of the Wnt signaling pathway. Neuronal apoptosis regulated by the cAMP/PKA signaling pathway is an important neuronal transduction pathway that plays an important role in the cognitive, learning, and memory functions of organisms [60]. Lidocaine has also been shown to improve CIRI in rats through the cAMP/PKA signaling pathway [60]. The cGMP-PKG pathway plays a key role in regulating cell growth, metabolism, and many other intracellular processes. The cGMP/PKG pathway in cardiomyocytes is inhibited by IR and preserved by ischemic post-regulation, which largely contributes to post-regulatory protection [61]. Combined with our findings, we conclude that luteolin may improve CIRI by regulating the calcium, PI3K-AKT, JAK-STAT, NF-kappa B, IL-17, cAMP, cGMP-PKG, and Wnt signaling pathways.

Finally, our RT-qPCR results showed that MCAO induction significantly up-regulated expression of the genes *Stoml3*, *Eomes*, and *Ms4a15*, while down-regulating *Nms*, *Ttr*, and *Avpr1a*; however, luteolin could partially reverse these changes in expression. In addition, the constructed PPI networks showed that *Ccl2* and *Angpt2* interacted with other DEGs. Stoml3 is necessary for normal mechanoreceptor function; as such targeting Stoml3 may be an effective way to reduce pain caused by harmful stress on bones and/or painful inflammatory pathology [62]. *Eomes* has been shown to positively correlate with the cytotoxic functions of cytotoxic CD4 T cells (CD4 CTLs). A previous study showed that *Eomes-*expressing CD4+ CTLs can act as biomarkers for the diagnosis and prognosis of secondary progressive multiple sclerosis with over 80% accuracy [63]. Another study suggested that *Eomes* is a potential biomarker for distinguishing radiation-induced brain injury-associated CD4 CTLs [64]. *Ms4a15* is an uncharacterized four-pass membrane protein, and its overexpression can significantly alter Ca^2+^ homeostasis and suppress IP_3_R1 expression, leading to extensive lipid remodeling [65]. Previous research has shown that *Nms* is down-regulated in experimental traumatic brain tissues, whereas progesterone can alleviate brain edema and induce an increase in *Nms* and its receptors [66]. Ttr is a tetramer transporter protein that plays a role in neuroprotection and promotes neurite growth in response to injury [67]. Gao et al. [68] previously demonstrated that *Avpr1a* was up-regulated following MCAO, and that modified constraint-induced movement therapy could down-regulate *Avpr1a* expression induced by MCAO. In addition, Ccl2, a member of the cytokine–cytokine receptor interaction family, plays a vital role in stroke pathophysiology [69]. Wu et al. [70] previously reported that Ccl2 exerted a critical effect on the blood–brain barrier integrity during CIRI. Angpt2, an angiogenic factor, is considered a promising biomarker for early screening of IS [71]. A previous study found that *Angpt2* could facilitate brain repair and attenuate cerebral ischemic injury via angiogenesis [72]. Based on these studies and our own results, we speculate that *Stoml3*, *Eomes*, *Ms4a15*, *Nms*, *Ttr*, *Avpr1a*, *Ccl2*, and *Angpt2* may mediate the beneficial effects of luteolin on CIRI; however, this remains to be verified in the future.

This study has some limitations which should be mentioned. First, the use of a single animal model and the potential variability in luteolin effects across different species or conditions should be acknowledged. Second, the specific mechanisms of pyroptosis in luteolin-induced CIRI should be investigated in detail. Additionally, the roles and exhaustive mechanisms of the identified DEGs (*Stoml3*, *Eomes*, *Ms4a15*, *Nms*, *Ttr*, *Avpr1a*, *Ccl2*, and *Angpt2*) as well as the enrichment of the calcium, PI3K-AKT, JAK-STAT, NF-kappa B, IL-17, cAMP, cGMP-PKG, and Wnt signaling pathways in CIRI progression need to be further verified through both *in vitro* and *in vivo* experiments.

## 5 Conclusion

Overall, the present study showed that luteolin alleviates brain infarction, apoptosis, and pyroptosis in MCAO-induced CIRI. Additionally, our analysis is the first to clarify the gene expression profiles of CIRI following luteolin treatment, from which it can be inferred that luteolin may improve MCAO-induced CIRI by targeting the identified DEGs (*Stoml3*, *Eomes*, *Ms4a15*, *Nms*, *Ttr*, *Avpr1a*, *Ccl2*, and *Angpt2*) and regulating the calcium, PI3K-AKT, JAK-STAT, NF-kappa B, IL-17, cAMP, cGMP-PKG, and Wnt signaling pathways. These findings provide a basis for the future comprehensive elucidation of the mechanisms underlying the role of luteolin in CIRI, and lay a theoretical foundation for the development of novel therapeutic targets and pathways for CIRI.

## Supplementary Material

Supplementary Table 1

Supplementary Table 2

Supplementary Table 3

Supplementary Table 4

Supplementary Table 5

Supplementary Table 6
